# Regeneration of the cerebral cortex by direct chemical reprogramming of macrophages into neuronal cells in acute ischemic stroke

**DOI:** 10.3389/fncel.2023.1225504

**Published:** 2023-08-11

**Authors:** Itaru Ninomiya, Akihide Koyama, Yutaka Otsu, Osamu Onodera, Masato Kanazawa

**Affiliations:** ^1^Department of Neurology, Brain Research Institute, Niigata University, Niigata, Japan; ^2^Department of Legal Medicine, Graduate School of Medical and Dental Science, Niigata University, Niigata, Japan

**Keywords:** direct reprogramming, small molecules, macrophage, neuron, stroke

## Abstract

Theoretically, direct chemical reprogramming of somatic cells into neurons in the infarct area represents a promising regenerative therapy for ischemic stroke. Previous studies have reported that human fibroblasts and astrocytes transdifferentiate into neuronal cells in the presence of small molecules without introducing ectopic transgenes. However, the optimal combination of small molecules for the transdifferentiation of macrophages into neurons has not yet been determined. The authors hypothesized that a combination of small molecules could induce the transdifferentiation of monocyte-derived macrophages into neurons and that the administration of this combination may be a regenerative therapy for ischemic stroke because monocytes and macrophages are directly involved in the ischemic area. Transcriptomes and morphologies of the cells were compared before and after stimulation using RNA sequencing and immunofluorescence staining. Microscopic analyses were also performed to identify cell markers and evaluate functional recovery by blinded examination following the administration of small molecules after ischemic stroke in CB-17 mice. In this study, an essential combination of six small molecules [CHIR99021, Dorsomorphin, Forskolin, isoxazole-9 (ISX-9), Y27632, and DB2313] that transdifferentiated monocyte-derived macrophages into neurons *in vitro* was identified. Moreover, administration of six small molecules after cerebral ischemia in model animals generated a new neuronal layer in the infarct cortex by converting macrophages into neuronal cells, ultimately improving neurological function. These results suggest that altering the transdifferentiation of monocyte-derived macrophages by the small molecules to adjust their adaptive response will facilitate the development of regenerative therapies for ischemic stroke.

## 1. Introduction

Ischemic stroke causes extensive neuronal death and, although antithrombotic agents and rehabilitation are the primary treatment options, their efficacy is limited (Hatakeyama et al., 2020; Otsu et al., 2020). As such, practical neuroregenerative technologies must be developed to achieve radical neurological improvement (Barker et al., 2018). Recently, direct chemical reprogramming using small molecules to transdifferentiate somatic cells into neurons has attracted considerable attention (Hu et al., 2015; Li et al., 2015; Gao et al., 2017; Xu et al., 2019; Yin et al., 2019). During the acute phase of ischemic stroke, circulating monocytes increase chemotaxis and accumulate in the infarct area, where they differentiate into macrophages involved in inflammation and remodeling (Shichita et al., 2012, 2017; Kanazawa et al., 2015). In contrast, we have reported that oxygen-glucose-deprived blood mononuclear cells converted to this phenotype and expressed pluripotent stem cell surface markers (Hatakeyama et al., 2019; Otsu et al., 2023). Monocytes and macrophages undergo transdifferentiation. Direct reprogramming of monocyte-derived macrophages into neuronal cells in vivo is theoretically possible with the administration of small-molecule drugs. However, the efficacy of this neuroregenerative therapy has not been verified because the optimal combination of chemical compounds that convert macrophages into neurons has not yet been determined. We hypothesized that a combination of small molecules could induce the transdifferentiation of monocyte-derived macrophages into neurons. Moreover, the administration of a combination of small molecules may be a regenerative therapy for ischemic stroke because monocytes and macrophages are directly involved in the ischemic area. As such, we investigated the possibility/feasibility of applying this technology to the treatment of ischemic stroke.

## 2. Methods

This study was conducted according to the principles of the Helsinki Declaration. Human blood samples were obtained with written informed consents from the healthy adults as approved by the Ethics Committee of the Genetic Analysis of Niigata University (approval number: G2020-215 0030). All animal protocols were approved by the Institutional Animal Care and Use Committee of Niigata University (approval number: SA00955).

### 2.1. Induction of human adult monocyte-derived macrophages

Peripheral blood mononuclear cells were isolated using density gradient centrifugation with Ficoll-Paque PREMIUM 1.073 (Cytiva) and SepMate-50 (Veritas) according to the manufacturer’s instructions. Freshly isolated peripheral blood mononuclear cells were seeded on plastic coverslips (Cell Desk LF, Sumitomo Bakelite) in an appropriate amount of monocyte attachment medium (PromoCell) in a density of 1 million/cm^2^ and incubated for 2 h at 5% CO2, 37°C. Subsequently, the non-adherent cells were removed by three washing steps with warm monocyte attachment medium. Adherent monocytes were cultured in macrophage medium, which consisted of X-VIVO 15 (Ronza) supplemented with 10% heat inactivated fetal bovine serum (FBS) (Biological Industries), and stimulated with 50 ng/ml macrophage colony-stimulating factor (Wako) for 7 days to differentiate them into monocyte-derived macrophages (Lescoat et al., 2018). We evaluated 15 different experiments (using five donors, three-time experiments).

### 2.2. Conversion of macrophages into neuronal cells

The conditioned media for neurodifferentiation consisted of Neurobasal (Gibco) supplemented with GlutaMAX, N2, and B27 supplements (0.5% each, Gibco), penicillin- streptomycin (Gibco), and brain-derived neurotrophic factor (BDNF) (20 ng/mL, Wako), glia-derived neurotrophic factor (GDNF) (20 ng/mL, Wako), neurotrophin-3 (NT3) (10 ng/mL, Wako), insulin-like growth factor 1 (IGF-1) (20 ng/mL, PEPROTECH), and basic fibroblast growth factor (bFGF) (20 ng/mL, Wako). Monocyte-derived macrophages were treated with the following chemical compounds: CHIR99021, 3 μM; Forskolin, 10 μM; Y-27632, 5 μM; Dorsomorphin, 2 μM; ISX-9, 3 μM; DB2313, 0.5 μM.

### 2.3. Conversion of macrophages into neuronal cells

Cells on plastic coverslips (Cell Desk LF, Sumitomo Bakelite) were fixed in 4% paraformaldehyde (PFA) (Wako) at room temperature for 10 min; subsequently PFA was washed off with phosphate-buffered saline (PBS) three times. After washing, the coverslips were transferred to blocking buffer and incubated for 1 h. The blocking buffer consisted of 1% bovine serum albumin and 0.1% Triton X-100 in PBS. After blocking, cells were incubated with primary antibodies (all 1:500) overnight at 4°C. On the second day, primary antibodies were washed off thrice with PBS. The cells were incubated with the corresponding secondary antibodies for 30 min at room temperature. The secondary antibodies were tagged with the fluorophores: Alexa Fluor 488 and Alexa 594 (1:1,000, Invitrogen). After incubation, the secondary antibodies were washed off thrice with PBS. Cells were mounted on glass slides with mounting solution containing 4′,6′-diamidino-2-phenylindole (DAPI; Vector Laboratories). Immunostaining results were analyzed using a fluorescence microscope (Olympus DSU). Confocal microscopy (ZEISS LSM710) was used to capture images. For each sample, the cells were counted at designated times in 10 randomly selected fields of view. The antibodies used are listed in Supplementary Table 1.

### 2.4. RNA extraction

Gene expression analysis was performed on the cultured cells. RNA was purified with the NucleoSpin^®^ RNA Plus kit (Macherey-Nagel) with initial quantitation conducted using a NanoDrop OneC spectrophotometer (Thermo Fisher Scientific). RNA quality was determined using a 2,200 Bioanalyzer (Agilent Technologies). Total RNA of the human cerebral cortex was obtained from Clontech (Cat. No. 636561).

### 2.5. Whole-transcript expression analysis using Clariom S

The purified RNA samples were subjected to whole transcriptomic analysis using the GeneChip Human Clariom S Array (Thermo Fisher Scientific; the analysis was outsourced to Life Technologies Japan Ltd.). The transcriptomic profiles of the cells were analyzed using Transcript Analysis Console 4.0.2 (TAC) software from Applied Biosystems. Gene ontology enrichment analysis was performed using the PANTHER program.^1^

### 2.6. Quantitative real-time polymerase chain reaction

The purified RNA samples were reverse-transcribed with SuperScript IV VILO Master Mix (Invitrogen) according to the manufacturer’s instructions. To select normalization factors, we used the free analysis program RefFinder^2^ to calculate the relative stability of the expression of 16 housekeeping genes (human housekeeping gene primer set; Takara) in each cultured cell. Two genes (RPLP2 and RPS18) were selected as controls for stable endogenous expression. Quantitative real-time polymerase chain reaction was conducted with 400 nM primers, 25 ng cDNA template, and TB Green Premix Ex Taq II (Takara) in a Thermal Cycler Dice Real-Time System (software version 5.11B for TP800, Takara) (*n* = 3). Data were log2 transformed to fit the normal distribution assumption of one-way analysis of variance (ANOVA). Primers used are listed in Supplementary Table 1.

### 2.7. Animals

Studies were conducted in adult CB-17/Icr-+/+Jcl (CB-17) mice aged 8 weeks (Clea Japan). The experimental animals had *ad libitum* access to food. Every effort was made to minimize the number of animals used and their suffering. Quantitative analyses were conducted by investigators blinded to the experimental protocol and sample identity.

### 2.8. Induction of ischemic stroke

Permanent focal cerebral ischemia was induced in mice by ligation and interruption of the distal portion of the left middle cerebral artery (MCA) as previously described (Nishie et al., 2021). In brief, under three types of mixed anesthesia, consisting of medetomidine, midazolam, and butorphanol, mice were placed in a lateral position. Under an operating microscope, an incision was made in the skin and the temporalis muscle was pushed aside to obtain a visual field for craniotomy. A burr hole was created in the skull using a drill. After carefully opening the dura mater, MCA occlusion (MCAO) was performed using electrocoagulation, followed by disconnection of the distal portion of the left MCA. The sham-operated mice underwent the same surgical procedure until the dura mater was opened.

### 2.9. Intraperitoneal injection of small molecules

A small molecules cocktail including CHIR99021, 10.54 μg/10 g; Dorsomorphin, 6.0 μg/10 g; Forskolin, 28.7 μg/10 g; Y27632, 23.7 μg/10 g; isoxazole-9 [ISX-9], 4.9 μg/10 g (Hu et al., 2015; Li et al., 2015; Gao et al., 2017; Yin et al., 2019); and DB2313, 2.5 μg/ mice weighted 10 g (Antony-Debré et al., 2017) or vehicle solution (dimethyl sulfoxide, DMSO 11.9 μL/ mice weighed 10 g) as control was injected into the peritoneum of mice daily starting 6 days after the induction of ischemic stroke. Fourteen days after the initial drug treatment, mice were sacrificed and transcardially perfused with 4% PFA. Whole brains were removed and post-fixed with 4% PFA for 24 h. Subsequently, the samples were embedded in paraffin and cut into 4 μm sections. The brain sections were subjected to immunohistochemistry using primary antibodies. Bound primary antibodies were visualized using Alexa Fluor 488 or 596 conjugated secondary antibodies (1:1,000, Invitrogen). Nuclei were counterstained with DAPI (Vector Laboratories). Images were acquired using an Olympus DSU or a Zeiss LSM 710 confocal microscope. The antibodies used are listed in Supplementary Table 1.

### 2.10. Behavioral tests

Phenotypic behavioral differences were assessed in sham-operated and MCAO mice in accordance with previous reports (Park et al., 2014; Tanaka et al., 2020), with minor modifications. Corner, wire hang, and basket tests were performed 2, 6, 13, and 20 days post stroke.

### 2.11. Corner test

A corner test was conducted using two wooden cardboards at an angle of 30° (Park et al., 2014; Hatakeyama et al., 2019). The mouse was made to enter the corner upon its placement midway to the corner. As the mouse reached deep into the corner, it turned backward toward the open end. The direction toward which the mouse turned was recorded. Twenty trials were recorded per animal on indicated days. The ischemic mice showed a marked preference for turning toward the non-impaired side (left turn). The percentage of left turns was analyzed as an indicator of deficit.

### 2.12. Wire hang test

The wire hang test was conducted using a square wire mesh plate (20 × 20 cm) made of warp and weft wires (0.8 mm in diameter) loosely woven in a mesh shape (Tanaka et al., 2020). Each mouse was placed on the wire mesh plate and allowed to acclimate to the environment for 10 s. The testing plate was then gently inverted and secured to the top of a cubic, open-topped glass box (25 × 25 × 25 cm). The latency to fall was measured.

### 2.13. Basket test

The basket test was conducted using a rectangular wire mesh basket (29 × 29 × 29.5 cm) (Tanaka et al., 2020). The basket was then gently inverted and placed on a flat plate where sawdust was spread. The latency to climb down the vertical walls of the basket to the flat plate was recorded, with a maximum trial time of 200 s. Three trials were completed for each mouse, with an interval of 5 min.

### 2.14. Statistical analysis

For comparison between two groups, statistical analysis was performed using the Student’s *t*-test. For comparisons among three or more groups, we used ANOVA followed by a *post-hoc* test (Tukey–Kramer test). All data are presented as the mean ± standard error of the mean (SEM).

## 3. Result

### 3.1. Small molecule candidates converted macrophages into neuronal cells

**FIGURE 1:**
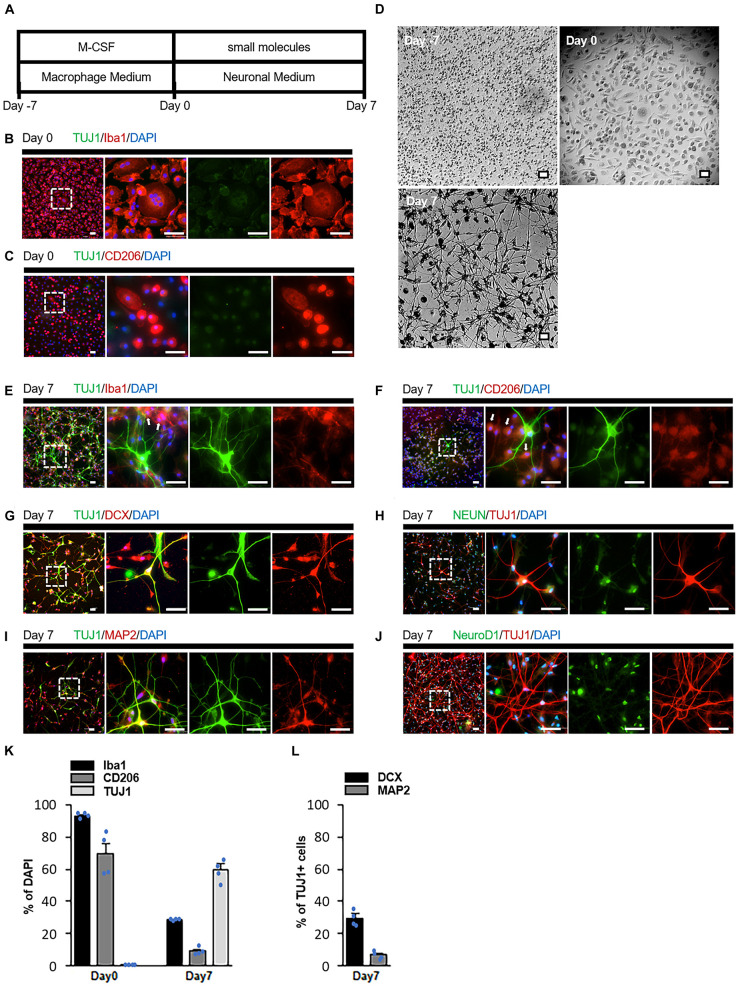
Transdifferentiation of human monocyte-derived macrophages into neuronal cells by small molecules. **(A)** Schematic diagram presenting the protocol of neuronal induction. **(B,C)** Immunostaining revealed that cultured cells (Day 0) expressed the macrophage markers Iba1 and CD206 but not the neuronal marker TUJ1. **(D)** Cells changed from monocytic (Day 7) and macrophage (Day 0) morphologies to neuronal (Day 7) morphology during induction (*n* = 15 from five donors, three-time experiments). **(E,F)** Immunostaining of cells treated with the small molecules. Some cells expressed the neuronal marker TUJ1, while others expressed the macrophage markers Iba1 and CD206 (arrow). **(G–J)** Immunostaining of small molecule-treated cells on Day 7 with TUJ1, DCX, NeuN, MAP2, and NeuroD1 antibodies. **(K,L)** Quantification of the conversion efficiency. Cells were calculated as the ratio of the number of Iba1, CD206 or TUJ1 positive cells to the total number of cells indicated by DAPI **(K)**. The number of cells was calculated as the ratio of the number of DCX or MAP2 positive cells to the TUJ1 positive cell number **(L)**. Data are represented as average ± SEM (*n* = 4 independent experiments). For comparison between two groups, statistical analysis was performed using Student’s *t*-test. Scale bar = 50 μm. IP, intraperitoneal; TUJ1, beta III tubulin; DCX, doublecortin; NeuN, neuronal nuclear protein; MAP2, microtubule-associated protein 2; DAPI, 4′,6′-diamidino-2-phenylindole; SEM, standard error of the mean.

We first examined small molecule candidates that chemically convert macrophages into neuronal cells (Figure 1A). Blood samples were obtained from healthy adults who provided informed consent, and peripheral blood mononuclear cells were isolated by centrifugation. Subsequently, the cells were cultured in monocyte attachment medium, and non-adherent cells were discarded. The remaining adherent monocytes were cultured in 10% FBS and 50 ng/ml recombinant human macrophage colony-stimulating factor. After 7 days, most cells were positive for macrophage markers Iba1 and CD206, indicating that monocyte-derived macrophages were induced (Figures 1B, C; Lescoat et al., 2018). We defined this time point as Day 0.

Day 0 macrophages were cultured with small molecules to investigate whether cells with a neuron-like morphology could be induced. CHIR99021, Dorsomorphin, Forskolin, ISX-9, and Y27632 were used as the small molecules. These compounds have been reported to be able to convert astrocytes and fibroblasts into neurons (Hu et al., 2015; Li et al., 2015; Yin et al., 2019). However, on Day 7, no cells presented neuron-like structures; rather, most of them transformed into cells with foamy structures (Supplementary Figure 1A1). Since the appearance of foam cells could morphologically indicate the activation of macrophages (Russell et al., 2009; Grajchen et al., 2018; Enos et al., 2019), we hypothesized that a chemical compound that silences macrophage specific genes is necessary for successful differentiation. PU.1 is one of the genes that play an important role in the differentiation of monocytes into macrophages (Klemsz et al., 1990; Zakrzewska et al., 2010). High levels of PU.1 expression in PU.1+/- fetal liver hematopoietic progenitors promoted macrophage differentiation (DeKoter and Singh, 2000). DB2313, a derivative of the heterocyclic diamidine family (Supplementary Figure 1B), efficiently inhibits PU.1 gene expression by an allosteric mechanism (Antony-Debré et al., 2017). Therefore, we cultured macrophages with a combination of six compounds: CDFIYB (CHIR99021, Dorsomorphin, Forskolin, ISX-9, Y27632, and DB2313). Some macrophages underwent drastic morphological changes and complex neurite-like structures were observed on Day 7 (Figure 1D).

Immunostaining indicated that these cells presented the neuronal markers doublecortin (DCX), beta-III tubulin (TUJ1), microtubule-associated protein 2 (MAP2), and neuronal nuclear protein (NeuN) (Figures 1E–I). However, these cells did not present the NueroD1 (Figure 1J). In contrast, some cells did not express any neuronal markers but remained positive for Iba1 and CD206 (Figures 1E, F). These results suggested that some macrophages that successfully differentiated into neurons and others that failed to differentiate existed simultaneously (Figure 1K). Among the TUJ1-positive cells, approximately 5% were MAP2-positive and 30% were DCX-positive, indicating that most of the induced neuronal cells were immature (Figure 1L). Conversion efficiency decreased when cells were cultured with all but one of the six compounds (Supplementary Figures 1 A1–A12, C). We defined cells treated with CHIR99021, Dorsomorphin, Forskolin, ISX-9, and Y27632 as Chemical Cocktail (CC) 5 cells, and those treated with these five compounds plus DB2313 as CC6 cells.

### 3.2. Transcriptome characteristics of induced neuronal cells

**FIGURE 2:**
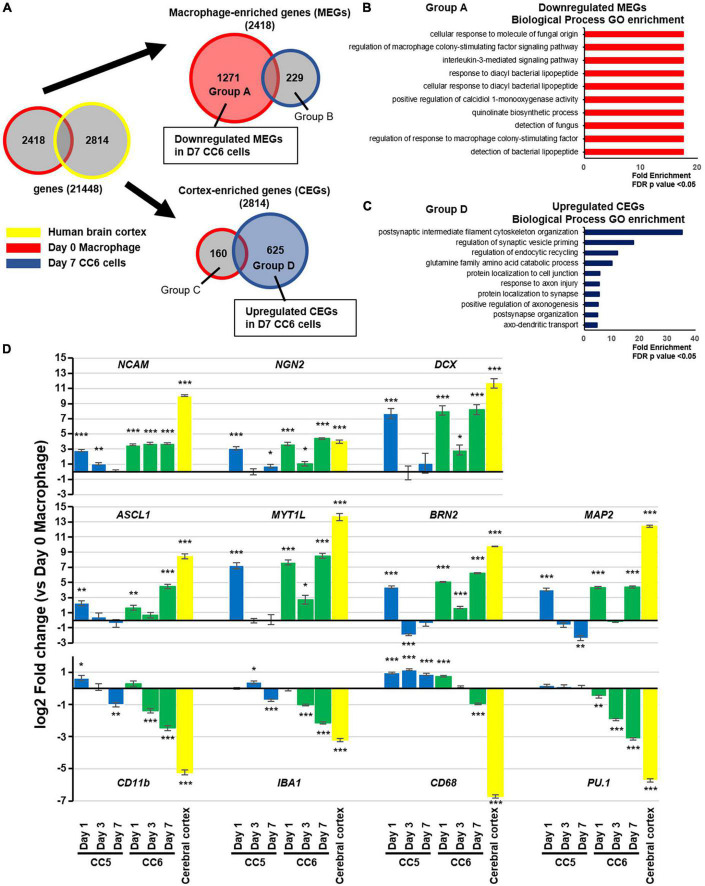
Transcriptome characteristics of induced neuronal cells. **(A)** Venn diagrams showing the protocol for microarray analysis. Comparing the human brain cortex and Day 0 macrophages, 2,814 genes (CEGs) were significantly expressed in the former and 2,418 (MEGs) in the latter. After 7 days of small molecule treatment, genes that were suppressed (1,271 genes) or enhanced (229 genes) among MEGs and genes that were suppressed (160 genes) or enhanced (625 genes) among CEGs were designated as groups A, B, C, and D, respectively. See also Supplementary Figures 2A–C. **(B)** Gene ontology enrichment analysis of the group A genes classified in panel **(A)**. **(C)** Gene ontology enrichment analysis of the group D genes classified in panel **(A)**. The enriched categories shown were based on a cutoff *p*-value of false discovery rate (FDR) < 0.05. **(D)** Real-time PCR validation of the expression of representative macrophage- and neuron enriched genes. Data were shown as fold change versus Day 0 macrophages. For comparisons among groups, we used ANOVA followed by a *post-hoc* test (Tukey–Kramer test). Mean ± SEM, *n* = 3 independent experiments, *p < 0.05, **p < 0.01, ***p < 0.001, versus Day 0 samples. CEG, cortex-enriched genes; MEG, macrophage-enriched genes; PCR, polymerase chain reaction; SEM, standard error of the mean.

We performed RNA microarray analysis on human brain cortex total RNA, Day 0 macrophages, Day 7 CC5 cells, and Day 7 CC6 cells. First, we compared the expression levels of 21,448 genes in the human brain cortex and Day 0 macrophages. Based on these results, we defined two groups of genes: cortex enriched genes (CEGs), whose expression was upregulated more than four-fold in the brain cortex, and macrophage-enriched genes (MEGs), whose expression was upregulated more than four-fold in Day 0 macrophages (Figure 2A and Supplementary Figure 2A). For these two groups of genes, we examined changes in gene expression levels from Day 0 macrophages to Day 7 CC6 cells. Of the 2,418 MEGs, the expression levels of 1,271 genes were suppressed to < one-half in Day 7 CC6 cells, and gene ontology analysis indicated significant enrichment of biological processes related to the interleukin-3-mediated signaling pathway, detection of fungus, and detection of bacterial lipopeptides, indicating that macrophage properties were repressed (Figure 2B and Supplementary Figure 2B). Of the total 2,814 CEGs, the expression levels of 625 genes were increased > two-fold in Day 7 CC6 cells, and gene ontology analysis indicated significant enrichment of biological processes related to positive regulation of axonogenesis, protein localization to synapse, and postsynaptic intermediate filament cytoskeleton organization, indicating that neural differentiation was induced (Figure 2C and Supplementary Figure 2C). Gene ontology analysis enrichment analysis of the group B genes and group B genes classified in Figure 2A was no regulation of neurons (Supplementary Figures 2D, E). Comparison of Day 7 CC5 cells with Day 0 macrophages exhibited no difference in the expression of CEGs such as *TUJ1* and *INA* and MEGs such as *PU.1* and *MAFB* (Supplementary Figure 2F).

Based on the results of the RNA microarray, we performed reverse transcription-quantitative polymerase chain reaction validation by comparing Day 0 macrophages, CC5 cells, and CC6 cells over time (Figure 2D). In CC6 cells, the expression of proneural genes such as *ASCL1* and *NGN2* and neuron-related genes increased over time, while the expression of macrophage related genes such as *CD11b*, *CD68*, and *PU.1* was suppressed. Interestingly, although CC5 cells demonstrated increased expression of proneural and neuron-related genes to the same extent as CC6 cells on Day 1, upregulation could not be maintained, and these genes were suppressed by Day 7. The reduction in the increase of CEGs on day 3 CC6 cells compared to Day 1 and Day 7 was observed. Macrophage-related genes were not sufficiently suppressed in CC5 cells throughout the study period. Finally, we performed RNA microarray analysis of Day 0 macrophages and Day 7 CC6 cells to determine whether the microglia were affected by the drug cocktail. The expression of all seven microglia-specific enriched genes was inhibited (all *P* < 0.001) (Supplementary Figure 2G).

### 3.3. Increased number of neurons in ischemic area after application of six chemical compounds

**FIGURE 3:**
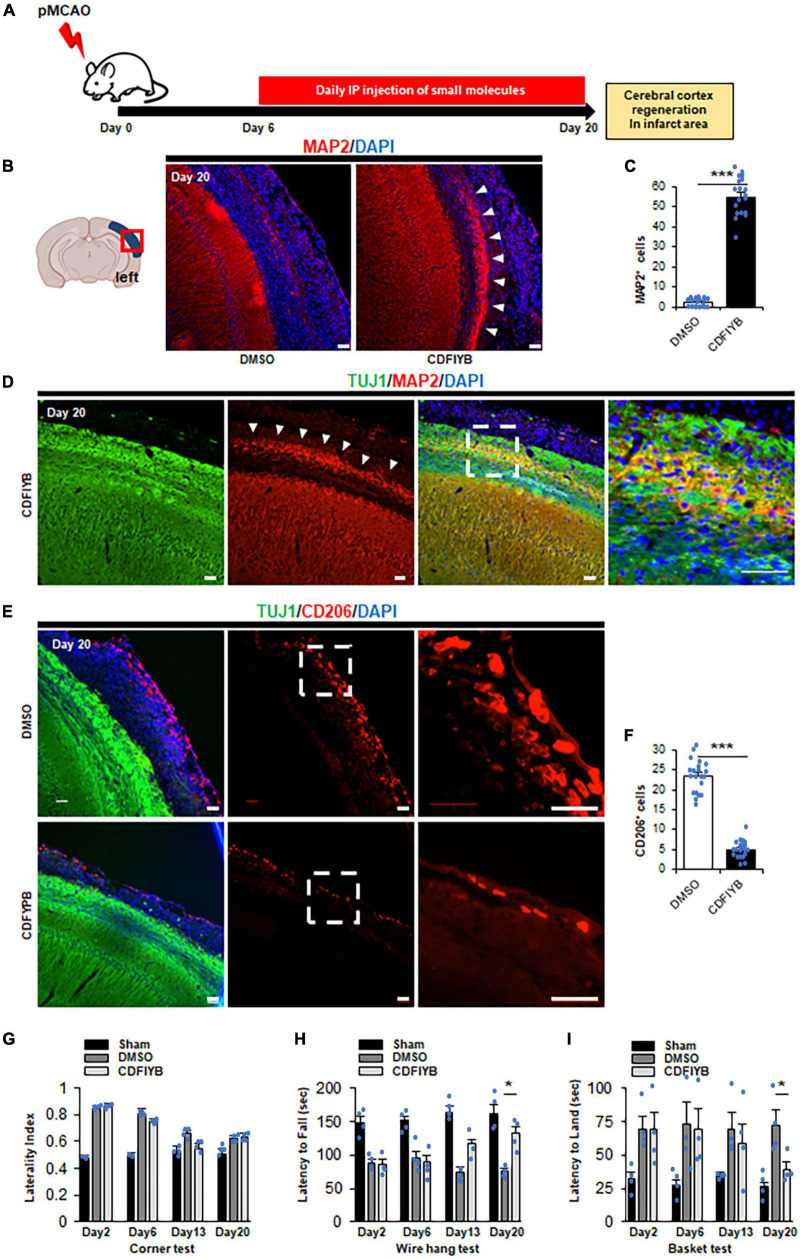
Significant increase of cortex neuron in ischemic area after application of six drugs. **(A)** Schematic diagram showing six drugs [CHIR99021, dorsomorphin, forskolin, isoxazole-9 (ISX-9), Y27632, DB2313] injected intraperitoneally daily for 14 days, 6 days after stroke onset. The mice were sacrificed at Day 20 for immunostaining. Representative images **(B)** and quantitative analyses **(C)** showing that compared to vehicle control by *t*-test, the six drugs significantly increased the neuronal cell number (arrowhead). Mean ± SEM, *n* = 3 independent experiments views. ***p < 0.001, Scale bar, 50 μm. The total number of MAP2+ cells in the infarct area was quantified based on six to seven randomly selected view fields in the infarct area for each sample. **(D)** Immunostaining against TUJ1 and MAP2 at Day 20. Arrowheads indicate a new neuronal layer that appeared in TUJ1+ ischemic regions after treatment with small molecules. Scale bars, 50 μm. Representative images of *n* = 3 independent experiments are shown. Representative images **(E)** and quantitative analyses **(F)** showing that six drugs significantly decreased the macrophage number compared to vehicle control by *t*-test. mean ± SEM, *n* = 3 independent experiments. ***p < 0.001, Scale bar, 50 μm. The total number of CD206+ cells in the infarct area was quantified based on six to seven randomly selected view fields in the infarct area for each sample. **(G–I)** Functional amylases by vehicle- and small molecule-treated mice on Day 20 by ANOVA *post hoc* Tukey–Kramer test (Mean ± SEM. *n* = 4) **(G)** The tendency to turn left at the corner did not change significantly between vehicle- and small molecule-treated mice. **(H)** The latency from the top of the basket d5own to the floor increased in the small molecule treated mice compared to the vehicle-treated mice. **(I)** The latency to fall from the mesh wire is decreased in the small molecule-treated mice compared to the vehicle-treated mice. *p < 0.05.

To investigate the therapeutic effects of these six drugs against stroke, we prepared a CB-17 wild-type mouse model of acute ischemic stroke. The left middle cerebral artery was permanently occluded in mice using electrocoagulation (Supplementary Figure 3A). Two days after the onset of ischemia, MAP2 immunoreactivity was markedly decreased in the left cerebral cortex, whereas TUJ1 immunoreactivity remained positive. We defined the MAP2-lost area as the infarct area (Popp et al., 2009). Twenty days after ischemia, the infarct cortical tissue gradually shrank, and TUJ1 immunoreactivity decreased in the outer cortex, dividing the infarct area into TUJ1-positive and negative areas (Supplementary Figure 3B). Macrophages accumulated more at the infarct area on Day 6 than on Day 2 (Supplementary Figure 3C), consistent with a previous report’s findings (Breckwoldt et al., 2008). We examined whether systemic administration of these six drugs could induce transdifferentiation of macrophages into neurons in the infarct area. Intraperitoneal injection of the six drug-cocktail CDFIYB was performed daily from days 6 to 20 (Figure 3A). Immunostaining of the brain tissues on Day 20 exhibited a new MAP2 positive layer that emerged in the infarct area, suggesting cortical regeneration (Figures 3B, C).

This newly generated cortical layer was confined to the TUJ1+ infarct area (Figure 3D). The number of Iba1+ or CD206+ cells in the infarct area significantly reduced, indicating that macrophages were consumed (Figures 3E, F and Supplementary Figures 4A, B). Although we investigated the possibility of small molecule cocktails which stimulate the activation of endogenous neural stem/progenitor cells (NSPCs) in the subventricular zone or dentate gyrus, there was no significant change in the number of DCX+ cells in the dentate gyrus in this study (Supplementary Figures 4C, D). We did not observe any morphological differences between sham animals and contralateral brains. Finally, in several assessments of motor sensory function, mice treated with small molecule compounds showed significant neurological improvement (Figures 3G–I). Additionally, adverse effects such as weight loss, decreased appetite, and liver or kidney dysfunction were not observed in mice administered DMSO or the six drugs.

## 4. Discussion

The present study demonstrated that administration of a combination of six molecules directly reprogrammed (i.e., transdifferentiated) monocyte-derived macrophages into neuronal cells. In particular, DB2313, a first-in-class small-molecule inhibitor of PU.1, was essential. Furthermore, when these six compounds were administered to mouse models of acute ischemic stroke, macrophages that accumulated in the infarct area were directly reprogrammed into neuronal cells *in vivo*, forming a newly generated cerebral cortex and improving neurological prognosis. Although it has been reported that neural stem/progenitor cells (NSPCs) endogenous to the subventricular zone or dentate gyrus supply some new neurons to the infarct area early after ischemic stroke onset (Arvidsson et al., 2002) and certain small molecule cocktails stimulate the activation of NSPCs *in vivo* (Yin et al., 2019). However, our results suggested that the newly generated cortical neurons were not derived from endogenous NSPCs by the small molecule cocktail. Thus, we confirmed the feasibility of developing small-molecule drugs that can convert macrophages into neurons, which will bring us one step closer to developing a novel neuroregenerative therapy for stroke.

Small molecules can directly convert fibroblasts and astrocytes into neuronal cells (Li et al., 2015; Gao et al., 2017). In clinical applications, however, frequent and large-scale harvesting of these cells is difficult due to the necessity of invasive biopsies. In contrast, large numbers of blood monocytes can be obtained via venipuncture, and the conversion of monocyte-derived macrophages into neurons enables the generation of human neuronal cells from any individual. In addition, unlike classic induced pluripotent stem (iPS) cell-based neuronal differentiation (Oki et al., 2012), our conversion method does not require the integration of ectopic transgenes and does not pass through the proliferative intermediate phase, thus preventing genomic gene modification and avoiding risks for tumorigenesis. Therefore, our approach can easily and safely obtain neuron-like cells for clinical applications.

From a technical perspective, cell culture for transdifferentiation often requires feeder cells such as glial cells and fibroblasts. Tanabe et al. (2018) reported that peripheral blood T cells can be converted into neurons using small molecules; however, this method requires fibroblasts as feeder cells. In contrast, the conversion of peripheral blood monocyte-derived macrophages into neurons using our method can be performed without feeder cells. Although feeder cell systems are useful for laboratory experiments, these support systems contain xenobiotic materials (increase the risk for biological pathogen cross-transfer). Our approach of using specific small molecules without feeder cells is superior to methods using feeder cells and is favorable for future clinical applications.

Although the small molecules CHIR99021, Dorsomorphin, Forskolin, ISX-9, and Y27632 used in this study have previously been reported to facilitate direct neural conversion (Hu et al., 2015; Li et al., 2015; Gao et al., 2017; Yin et al., 2019), the combination of CHIR99021, Dorsomorphin, Forskolin, ISX-9, and Y27632 was novel efficiency for monocyte transdifferentiation. Specifically, CHIR99021 enhance transcription factors-mediated neuronal conversion efficiency (Hu et al., 2015) and was essential for chemical-mediated neuronal conversion in adult astrocytes (Gao et al., 2017). Dorsomorphin may be involved in promoting neuronal specification or maturation of induced neuronal cells in combination with the transforming growth factor-beta signaling pathway (Hong and Yu, 2009; Hu et al., 2015). Forskolin reduces lipid peroxidation, promotes neuronal conversion efficiency (Liu et al., 2013; Gascón et al., 2016), and induces changes in cell morphology (Gao et al., 2017). ISX-9 activated neuronal genes (Gao et al., 2017). Y27632 facilitates the neuronal conversion of human fibroblasts and results in the generation of TUJ1-positive cells with neuron-like morphology (Hu et al., 2015). In this study, we added DB2313, which efficiently inhibited the expression of the macrophage marker PU.1 (Antony-Debré et al., 2017). For neuronal transdifferentiation, inhibition of the *PU.1* gene was efficient in macrophages. Overexpression of PU.1 efficiently reprogramed neural stem cells to monocytes (Forsberg et al., 2010). We speculate that DB2313 plays a key role in the inhibition of lymphoid and myeloid cell fates. If PU.1 was expressed, the macrophages would be in a steady state trying to maintain their nature, and neural induction is not likely to be successful. Transient changes in gene expression are common in various cellular processes such as development, differentiation, and response to environmental cues. They play crucial roles in maintaining cellular homeostasis and in adapting to changing conditions. Proneural gene expression is suppressed to maintain macrophage function. Therefore, the Day 3 response may be negative feedback. In the early stage of direct reprogramming into neurons, the majority of cells first express neuron-related genes in response to stimuli. However, in the intermediate stage, perhaps due to epigenetic barriers, the cells fail to acquire a permanent neuron-like identity and revert to their original cell type or diverge to an alternative fate that is neither neuronal nor original if there is no appropriate gene expression control for the cells (Treutlein et al., 2016). DB2313 is essential for macrophages to overcome one of these barriers. These results suggested that the specific combination of small molecules required for neural conversion varies depending on the source cell type.

The present study had several limitations. First, the conversion efficacy and neuronal purity of TUJ1/DCX double-positive cells was low (approximately 30%). These cells were less mature than mouse fibroblasts or iPS cell-derived neuronal cells (Kim et al., 2011; Matsui et al., 2012; Zhang et al., 2013). Second, the long-term effects of administration of small molecules are unclear. After cerebral ischemia, the transplanted iPS cells stopped proliferating but survived without forming tumors for at least 4 months and differentiated into morphologically mature neurons of different subtypes (Oki et al., 2012). Notwithstanding, the long-term effects of administration of small molecules must be carefully evaluated. We did not evaluate the exact concentration of each drug in the brain parenchyma. Therefore, it is difficult to ensure that all the six drugs reach the brain sufficiently to exert their effects. Determining the brain concentration of each drug is necessary. Moreover, this combination of compounds did not affect the migration of macrophages into the brain. Previous reports indicate that in the event of an ischemic stroke, the number of infiltrated macrophages peaks at 48–72 h after onset (Mabuchi et al., 2000; Liu and Sorooshyari, 2021). We administered drugs after Day 6. Therefore, we speculate that the effects of the infiltrating monocytes and macrophages in the brain after treatment were small in this study. However, we cannot eliminate the possibility that the beneficial effects, leading to improved motor functions, might not be attributed to the anti-inflammatory mechanisms. Rather, these drugs probably promote regeneration by inducing transdifferentiation of macrophages. Finally, CHIR99021, dorsomorphin, forskolin, ISX-9, Y27632 can induce the differentiation of fibroblasts and astrocytes into neurons (Hu et al., 2015; Li et al., 2015; Gao et al., 2017; Yin et al., 2019). Moreover, our drug cocktail could affect microglia *in vivo* because of inhibition of microglia -specific enriched genes. We could not determine how much of the new MPA2 positive layer was due to differentiated astrocytes, fibroblasts, or microglia.

## 5. Conclusion

In conclusion, peripheral blood monocyte-derived macrophages might be differentiated into neurons. Although data from future studies are required to support the concept of cell transdifferentiation, the transdifferentiation of monocyte-derived macrophages by the small molecules to adjust their adaptive responses will facilitate the development of regenerative therapies for ischemic stroke.

## Data availability statement

The datasets presented in this study can be found in online repositories. The names of the repository/repositories and accession number(s) can be found below: https://www.ncbi.nlm.nih.gov/, GSE214644.

## Ethics statement

The studies involving humans were approved by The Ethics Committee of the Genetic Analysis of Niigata University. The studies were conducted in accordance with the local legislation and institutional requirements. The participants provided their written informed consent to participate in this study. The animal study was approved by The Institutional Animal Care and Use Committee of Niigata University. The study was conducted in accordance with the local legislation and institutional requirements.

## Author contributions

IN: conceptualization and writing–original draft. IN and AK: methodology. IN, YO, and AK: investigation. IN, AK, YO, OO, and MK: writing–review and editing. IN, OO, and MK: resources. MK: supervision. All authors contributed to the article and approved the submitted version.

## Footnotes

1
http://geneontology.org/

2
https://blooge.cn/RefFinder/

## References

[B1] Antony-Debré I. Paul A. Leite J. Mitchell K. Kim H. Carvajal L. (2017). Pharmacological inhibition of the transcription factor PU.1 in leukemia. *J. Clin. Invest.* 127 4297–4313. 10.1172/JCI92504 29083320 PMC5707147

[B2] Arvidsson A. Collin T. Kirik D. Kokaia Z. Lindvall O. (2002). Neuronal replacement from endogenous precursors in the adult brain after stroke. *Nat. Med.* 8 963–970. 10.1038/nm747 12161747

[B3] Barker R. Götz M. Parmar M. (2018). New approaches for brain repair-from rescue to reprogramming. *Nature* 557 329–334. 10.1038/s41586-018-0087-1 29769670

[B4] Breckwoldt M. Chen J. Stangenberg L. Aikawa E. Rodriguez E. Qiu S. (2008). Tracking the inflammatory response in stroke in vivo by sensing the enzyme myeloperoxidase. *Proc. Natl. Acad. Sci. U.S.A.* 105 18584–18589. 10.1073/pnas.0803945105 19011099 PMC2587593

[B5] DeKoter R. Singh H. (2000). Regulation of B lymphocyte and macrophage development by graded expression of PU.1. *Science* 288 1439–1441. 10.1126/science.288.5470.1439 10827957

[B6] Enos N. Takenaka H. Scott S. Salfity H. Kirk M. Egar M. (2019). Meningeal foam cells and ependymal cells in axolotl spinal cord regeneration. *Front. Immunol.* 10:2558. 10.3389/fimmu.2019.02558 31736973 PMC6838144

[B7] Forsberg M. Carlén M. Meletis K. Yeung M. Barnabé-Heider F. Persson M. (2010). Efficient reprogramming of adult neural stem cells to monocytes by ectopic expression of a single gene. *Proc. Natl. Acad. Sci. U.S.A.* 107 14657–14661. 10.1073/pnas.1009412107 20675585 PMC2930460

[B8] Gao L. Guan W. Wang M. Wang H. Yu J. Liu Q. (2017). Direct generation of human neuronal cells from adult astrocytes by small molecules. *Stem Cell Rep.* 8 538–547. 10.1016/j.stemcr.2017.01.014 28216149 PMC5355633

[B9] Gascón S. Murenu E. Masserdotti G. Ortega F. Russo G. Petrik D. (2016). Identification and successful negotiation of a metabolic checkpoint in direct neuronal reprogramming. *Cell Stem Cell* 18 396–409. 10.1016/j.stem.2015.12.003 26748418

[B10] Grajchen E. Hendriks J. Bogie J. (2018). The physiology of foamy phagocytes in multiple sclerosis. *Acta Neuropathol. Commun.* 6:124. 10.1186/s40478-018-0628-8 30454040 PMC6240956

[B11] Hatakeyama M. Kanazawa M. Ninomiya I. Omae K. Kimura Y. Takahashi T. (2019). A novel therapeutic approach using peripheral blood mononuclear cells preconditioned by oxygen-glucose deprivation. *Sci. Rep.* 9:16819. 10.1038/s41598-019-53418-5 31728010 PMC6856386

[B12] Hatakeyama M. Ninomiya I. Otsu Y. Omae K. Kimura Y. Onodera O. (2020). Cell therapies under clinical trials and polarized cell therapies in pre-clinical studies to treat ischemic stroke and neurological diseases: A literature review. *Int. J. Mol. Sci.* 21:6194. 10.3390/ijms21176194 32867222 PMC7503631

[B13] Hong C. Yu P. (2009). Applications of small molecule BMP inhibitors in physiology and disease. *Cytokine Growth Factor Rev.* 20 409–418. 10.1016/j.cytogfr.2009.10.021 19914855 PMC2813719

[B14] Hu W. Qiu B. Guan W. Wang Q. Wang M. Li W. (2015). Direct conversion of normal and Alzheimer’s disease human fibroblasts into neuronal cells by small molecules. *Cell Stem Cell* 17 204–212. 10.1016/j.stem.2015.07.006 26253202

[B15] Kanazawa M. Kawamura K. Takahashi T. Miura M. Tanaka Y. Koyama M. (2015). Multiple therapeutic effects of progranulin on experimental acute ischaemic stroke. *Brain* 138 1932–1948. 10.1093/brain/awv079 25838514

[B16] Kim J. Efe J. Zhu S. Talantova M. Yuan X. Wang S. (2011). Direct reprogramming of mouse fibroblasts to neural progenitors. *Proc. Natl. Acad. Sci. U.S.A.* 108 7838–7843. 10.1073/pnas.1103113108 21521790 PMC3093517

[B17] Klemsz M. McKercher S. Celada A. Van Beveren C. Maki R. (1990). The macrophage and B cell-specific transcription factor PU.1 is related to the ets oncogene. *Cell* 183 113–124. 10.1016/0092-8674(90)90219-5 2180582

[B18] Lescoat A. Ballerie A. Augagneur Y. Morzadec C. Vernhet L. Fardel O. (2018). Distinct properties of human M-CSF and GM-CSF monocyte-derived macrophages to simulate pathological lung conditions in vitro: Application to systemic and inflammatory disorders with pulmonary involvement. *Int. J. Mol. Sci.* 19:894. 10.3390/ijms19030894 29562615 PMC5877755

[B19] Li X. Zuo X. Jing J. Ma Y. Wang J. Liu D. (2015). Small-molecule-driven direct reprogramming of mouse fibroblasts into functional neurons. *Cell Stem Cell* 17 195–203. 10.1016/j.stem.2015.06.003 26253201

[B20] Liu M. Zang T. Zou Y. Chang J. Gibson J. Huber K. (2013). Small molecules enable neurogenin 2 to efficiently convert human fibroblasts into cholinergic neurons. *Nat. Commun.* 4:2183. 10.1038/ncomms3183 23873306 PMC3843951

[B21] Liu Q. Sorooshyari S. (2021). Quantitative and correlational analysis of brain and spleen immune cellular responses following cerebral ischemia. *Front. Immunol.* 12:617032. 10.3389/fimmu.2021.617032 34194419 PMC8238006

[B22] Mabuchi T. Kitagawa K. Ohtsuki T. Kuwabara K. Yagita Y. Yanagihara T. (2000). Contribution of microglia/macrophages to expansion of infarction and response of oligodendrocytes after focal cerebral ischemia in rats. *Stroke* 31 1735–1743. 10.1161/01.str.31.7.1735 10884481

[B23] Matsui T. Takano M. Yoshida K. Ono S. Fujisaki C. Matsuzaki Y. (2012). Neural stem cells directly differentiated from partially reprogrammed fibroblasts rapidly acquire gliogenic competency. *Stem Cells* 30 1109–1119. 10.1002/stem.1091 22467474

[B24] Nishie H. Nakano-Doi A. Sawano T. Nakagomi T. (2021). Establishment of a reproducible ischemic stroke model in nestin-GFP mice with high survival rates. *Int. J. Mol. Sci.* 22:12997. 10.3390/ijms222312997 34884811 PMC8657611

[B25] Oki K. Tatarishvili J. Wood J. Koch P. Wattananit S. Mine Y. (2012). Human-induced pluripotent stem cells form functional neurons and improve recovery after grafting in stroke-damaged brain. *Stem Cells* 30 1120–1133. 10.1002/stem.1104 22495829

[B26] Otsu Y. Hatakeyama M. Kanayama T. Akiyama N. Ninomiya I. Omae K. (2023). Oxygen-glucose deprived peripheral blood mononuclear cells protect against ischemic stroke. *Neurotherapeutics* 10.1007/s13311-023-01398-w 37335500 PMC10480381

[B27] Otsu Y. Namekawa M. Toriyabe M. Ninomiya I. Hatakeyama M. Uemura M. (2020). Strategies to prevent hemorrhagic transformation after reperfusion therapies for acute ischemic stroke: A literature review. *J. Neurol. Sci.* 419:117217. 10.1016/j.jns.2020.117217 33161301

[B28] Park S. Marasini S. Kim G. Ku T. Choi C. Park M. (2014). A method for generating a mouse model of stroke: Evaluation of parameters for blood flow, behavior, and survival [corrected]. *Exp. Neurobiol.* 23 104–114. 10.5607/en.2014.23.1.104 24737945 PMC3984953

[B29] Popp A. Jaenisch N. Witte O. Frahm C. (2009). Identification of ischemic regions in a rat model of stroke. *PLoS One* 4:e4764. 10.1371/journal.pone.0004764 19274095 PMC2652027

[B30] Russell D. Cardona P. Kim M. Allain S. Altare F. (2009). Foamy macrophages and the progression of the human tuberculosis granuloma. *Nat. Immunol.* 10 943–948. 10.1038/ni.1781 19692995 PMC2759071

[B31] Shichita T. Hasegawa E. Kimura A. Morita R. Sakaguchi R. Takada I. (2012). Peroxiredoxin family proteins are key initiators of post-ischemic inflammation in the brain. *Nat. Med.* 18 911–917. 10.1038/nm.2749 22610280

[B32] Shichita T. Ito M. Morita R. Komai K. Noguchi Y. Ooboshi H. (2017). MAFB prevents excess inflammation after ischemic stroke by accelerating clearance of damage signals through MSR1. *Nat. Med.* 23 723–732. 10.1038/nm.4312 28394332

[B33] Tanabe K. Ang C. Chanda S. Olmos V. Haag D. Levinson D. (2018). Transdifferentiation of human adult peripheral blood T cells into neurons. *Proc. Natl. Acad. Sci. U.S.A.* 115 6470–6475. 10.1073/pnas.1720273115 29866841 PMC6016798

[B34] Tanaka Y. Nakagomi N. Doe N. Nakano-Doi A. Sawano T. Takagi T. (2020). Early reperfusion following ischemic stroke provides beneficial effects, even after lethal ischemia with mature neural cell death. *Cells* 9:1374. 10.3390/cells9061374 32492968 PMC7349270

[B35] Treutlein B. Lee Q. Camp J. Mall M. Koh W. Shariati S. (2016). Dissecting direct reprogramming from fibroblast to neuron using single-cell RNA-seq. *Nature* 534 391–395. 10.1038/nature18323 27281220 PMC4928860

[B36] Xu G. Wu F. Gu X. Zhang J. You K. Chen Y. (2019). Direct conversion of human urine cells to neurons by small molecules. *Sci. Rep.* 9:16707. 10.1038/s41598-019-53007-6 31723223 PMC6854089

[B37] Yin J. Zhang L. Ma N. Wang Y. Lee G. Hou X. (2019). Chemical conversion of human fetal astrocytes into neurons through modulation of multiple signaling pathways. *Stem Cell Rep.* 12 488–501. 10.1016/j.stemcr.2019.01.003 30745031 PMC6409415

[B38] Zakrzewska A. Cui C. Stockhammer O. Benard E. Spaink H. Meijer A. (2010). Macrophage-specific gene functions in Spi1-directed innate immunity. *Blood* 116 e1–e11. 10.1182/blood-2010-01-262873 20424185

[B39] Zhang Y. Pak C. Han Y. Ahlenius H. Zhang Z. Chanda S. (2013). Rapid single-step induction of functional neurons from human pluripotent stem cells. *Neuron* 78 785–798. 10.1016/j.neuron.2013.05.029 23764284 PMC3751803

