# Corrigendum: Regeneration of the cerebral cortex by direct chemical reprogramming of macrophages into neuronal cells in acute ischemic stroke

**DOI:** 10.3389/fncel.2024.1372045

**Published:** 2024-02-05

**Authors:** Itaru Ninomiya, Akihide Koyama, Yutaka Otsu, Osamu Onodera, Masato Kanazawa

**Affiliations:** ^1^Department of Neurology, Brain Research Institute, Niigata University, Niigata, Japan; ^2^Department of Legal Medicine, Graduate School of Medical and Dental Science, Niigata University, Niigata, Japan

**Keywords:** direct reprogramming, small molecules, macrophage, neuron, stroke

In the published article, a section of text was mistakenly not included in the publication. The missing section appears below:

## 2.2 Conversion of macrophages into neuronal cells

The conditioned media for neurodifferentiation consisted of Neurobasal (Gibco) supplemented with GlutaMAX, N2, and B27 supplements (0.5% each, Gibco), penicillin-streptomycin (Gibco), and brain-derived neurotrophic factor (BDNF) (20 ng/mL, Wako), glia-derived neurotrophic factor (GDNF) (20 ng/mL, Wako), neurotrophin-3 (NT3) (10 ng/mL, Wako), insulin-like growth factor 1 (IGF-1) (20 ng/mL, PEPROTECH), and basic fibroblast growth factor (bFGF) (20 ng/mL, Wako). Monocyte-derived macrophages were treated with the following chemical compounds: CHIR99021, 3 μM; Forskolin, 10 μM; Y-27632, 5 μM; Dorsomorphin, 2 μM; ISX-9, 3 μM; DB2313, 0.5 μM.

The authors apologize for this error and state that this does not change the scientific conclusions of the article in any way. The original article has been updated.

