# Supplementary material for: Regeneration of the cerebral cortex by direct chemical reprogramming of macrophages into neuronal cells in acute ischemic stroke
Source: Front Cell Neurosci. 2023 Aug 11;17:1225504. doi: 10.3389/fncel.2023.1225504 (PMC10457112; doi:10.3389/fncel.2023.1225504)
Supplement: Supplementary file 1 [file Data_Sheet_1.PDF]

Supplementary Material

Regeneration of the cerebral cortex by direct chemical reprogramming of macrophages into neuronal cells in acute ischemic stroke

Itaru Ninomiya[1*], Akihide Koyama[2], Yutaka Otsu[1], Osamu Onodera[1], Masato Kanazawa[1*]

[1]Brain Research Institute, Niigata University, Department of Neurology, Niigata, Japan

[2]Graduate School of Medical and Dental Science, Niigata University, Department of Legal Medicine, Niigata, Japan

*** Correspondence:**
Itaru Ninomiya: ininomiya@bri.niigata-u.ac.jp

Masato Kanazawa: masa2@bri.niigata-u.ac.jp

1 Supplementary Table 1 Related to Experimental Procedures. Antibodies and Oligonucleotides

RESOURCE	SOURCE	IDENTIFIER
Antibodies		
Polyclonal rabbit anti-beta III Tubulin (TUJ1)	Abcam	Cat#ab18207; RRID:AB_444319
Monoclonal mouse anti-TUJ1	BioLegend	Cat#801201; RRID:AB_ 2313773
Polyclonal rabbit anti-Doublecortin (DCX)	Abcam	Cat#ab18723; RRID:AB_732011
Monoclonal mouse anti-DCX	Santa Cruz	Cat#sc-271390; RRID:AB_10610966
Polyclonal rabbit anti-Microtubule-Associated Protein 2 (MAP2)	Millipore	Cat#AB5622;<br>RRID:AB_91939

Monoclonal mouse anti-MAP 2	Sigma-Aldrich	Cat#M4403;<br>RRID:AB_477193
Monoclonal rabbit anti-NeuN	Abcam	Cat# ab177487;<br>RRID:AB_2532109
Monoclonal mouse anti-NeuroD1	Abcam	Cat#ab60704;<br>RRID:AB_ 943491
Polyclonal rabbit anti-Iba1	Proteintech	Cat#10904-1-AP;<br>RRID:AB_2224377
Polyclonal Rabbit anti-CD206	Abcam	Cat#ab64693;<br>RRID:AB_1523910
Oligonucleotides		
Primer for MAP2 F:<br>GTCACTTGCAACCAGAAATTGGA	Takara Bio Inc.	SetID:HA268080-F
Primer for MAP2 R:<br>GCTGCTGGAACTCAGCAGGTAA	Takara Bio Inc.	SetID:HA268080-R
Primer for DCX F:<br>CGGAAGCATGGATGAACTGG	Takara Bio Inc.	SetID:HA156128-F
Primer for DCX R:<br>CAGTTGGGATTGACATTCTTGGTG	Takara Bio Inc.	SetID:HA156128-R
Primer for MYT1L F:<br>CCGTACGGAGGTGTGCAATTC	Takara Bio Inc.	SetID:HA281776-F
Primer for MYT1L R:<br>GCAACATTATTGATCAGCCGTGAG	Takara Bio Inc.	SetID:HA281776-R

Primer for ASCL1 F: GTCACAAGTCAGCGCCCAAG	Takara Bio Inc.	SetID:HA140715-F
Primer for ASCL1 R: TGTAGCCAAAGCCGCTGAAG	Takara Bio Inc.	SetID:HA140715-R
Primer for BRN2 F: ACACTGACGATCTCCACGCAGTA	Takara Bio Inc.	SetID:HA152014-F
Primer for BRN2 R: GAGGGTGTGGGACCCTAAATATGAC	Takara Bio Inc.	SetID:HA152014-R
Primer for NGN2 F: GGCACAGGCCAAAGTCACAG	Takara Bio Inc.	SetID:HA144604-F
Primer for NGN2 R: CGATCCGAGCAGCACTAACAC	Takara Bio Inc.	SetID:HA144604-R
Primer for CD11b F: GCTGCCGGTGAAATATGCTG	Takara Bio Inc.	SetID:HA261098-F
Primer for CD11b R: TCTCTGAGGCCGTGAAGTTGA	Takara Bio Inc.	SetID:HA261098-R
Primer for Iba1 F: CAGGATGATGCTGGGCAAGA	Takara Bio Inc.	SetID:HA298334-F
Primer for Iba1 R: CCTTCAAATCAGGGCAACTCAGA	Takara Bio Inc.	SetID:HA298334-R
Primer for NCAM F: AATTTACCGCGGCAAGAACATC	Takara Bio Inc.	SetID:HA278033-F
Primer for NCAM R: CCTGGCTGGGAACAATATCCAC	Takara Bio Inc.	SetID:HA278033-R

Primer for PU.1 F: GCCCTATGACACGGATCTATACCAA	Takara Bio Inc.	SetID:HA102872-F
Primer for PU.1 R: TCTCGGCGAAGCTCTCGAA	Takara Bio Inc.	SetID:HA102872-R
Primer for CD68 F: AGCAGGGTTGAGCAACTGGTG	Takara Bio Inc.	SetID:HA070317-F
Primer for CD68 R: AGCCAGCCTCATGGCTGAA	Takara Bio Inc.	SetID:HA070317-R

2 Supplementary Figure 1

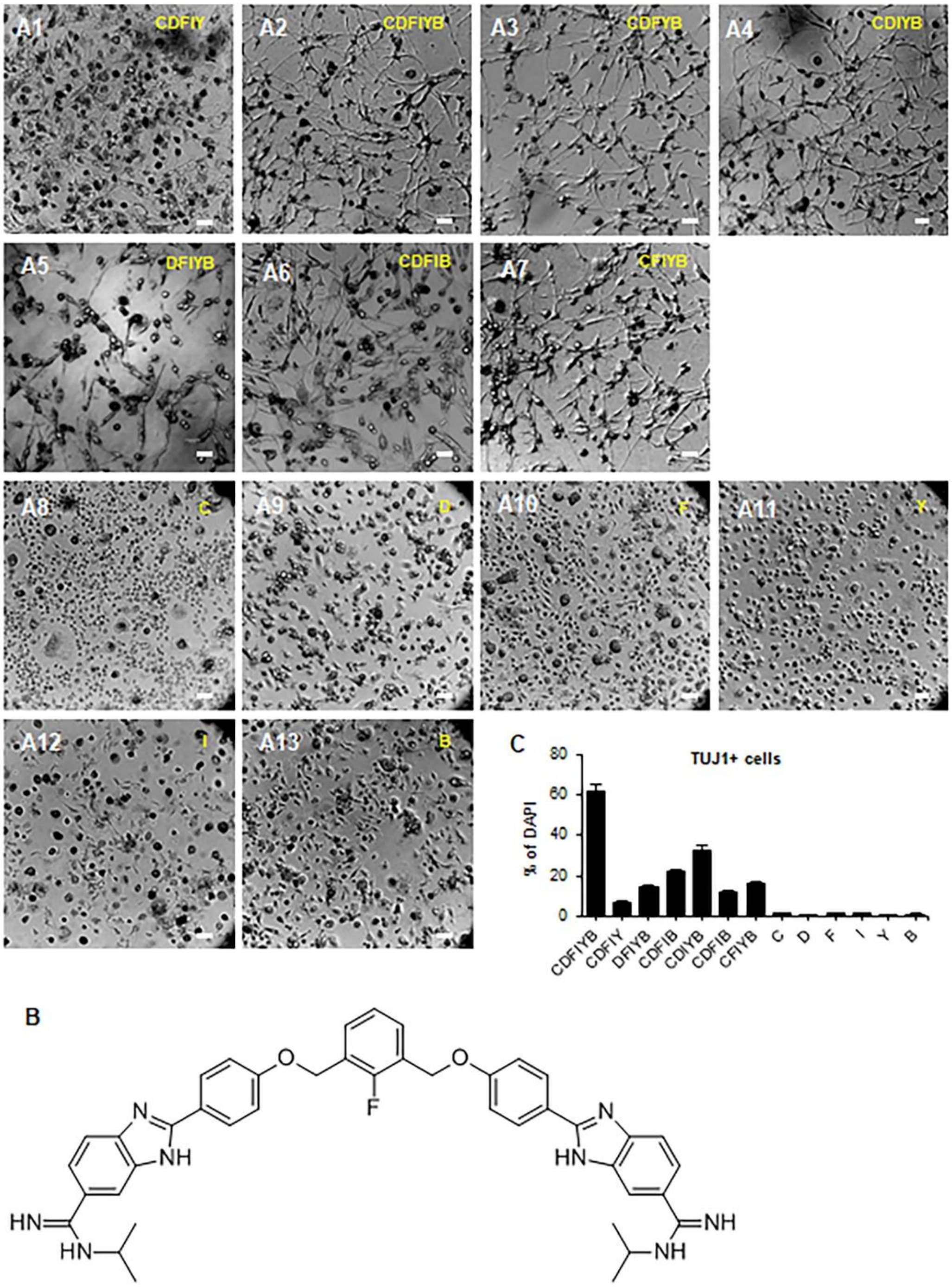

Supplementary Figure 1. Further confirmation of neuronal conversion. Related to Figures 1. (**A1-A13**) Representative morphological images of cells treated with the indicated small molecule(s) for 7 days. Yellow letters in the upper-right corner of each panel indicate small molecule(s). C, D, F, I, Y, and B, represent CHIR99021, Dorsomorphin, Forskolin, isoxazole-9 (ISX-9), 439 Y27632, and DB2313, respectively. Representative images of three independent experiments are shown. Scale bar = 50 µm. (**B**) Chemical structures of DB2313. (**C**) Quantification of conversion efficiency on Day 7 using the indicated small molecule(s). Mean ± SEM, n = 3 independent experiments. SEM, standard error of mean.

3 Supplementary Figure 2

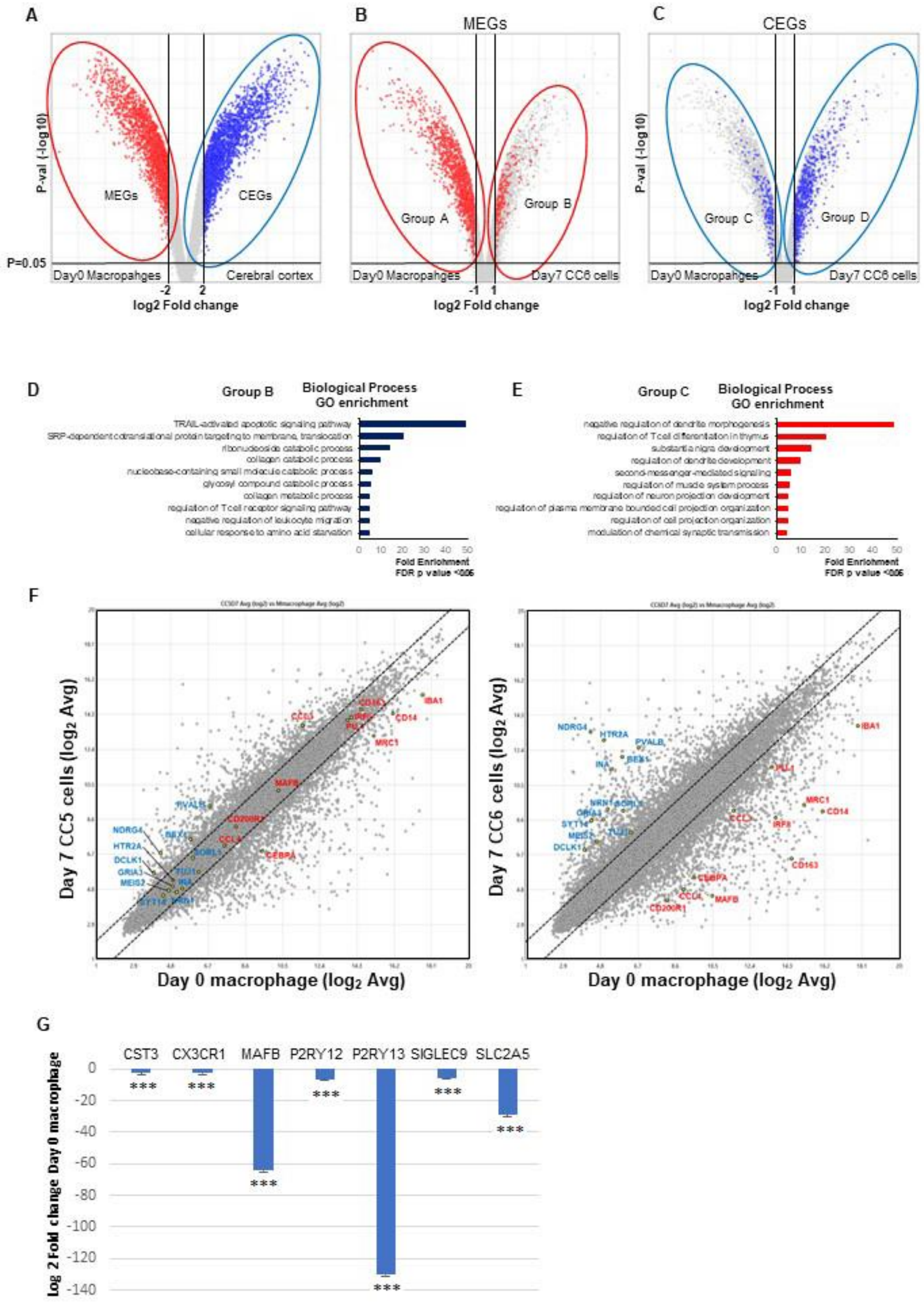

Supplementary Figure 2. Further analysis of neuronal conversion process. Related to Figure 2. (**A**) Volcano plot of 21448 genes with log2 ratio of expression levels of human cerebral cortex/Day-0-macrophages (x-axis) and the –log10 of the corresponding significance value (p value, y-axis); four-fold changes (vertical lines), significance cutoff p = 0.05 (horizontal line). Genes significantly enhanced in the cerebral cortex are shown in blue (CEGs) and those enhanced in Day 0 macrophages (MEGs) are shown in red. (**B**) Volcano plot of MEGs (2418 genes) with log2 ratio of expression levels of Day 7-CC6-cells/Day 0-macrophages (x-axis) and –log10 of the corresponding significance value (p value, y-axis); two-fold changes (vertical lines), significance cutoff p = 0.05 (horizontal line). (**C**) Volcano plot of CEGs (2148 genes) with log2 ratio of expression levels of Day 7-CC6-cells/Day 0-macrophages (x-axis) and –log10 of the corresponding significance value (p value, y-axis); two-fold changes (vertical lines), significance cutoff p = 0.05 (horizontal line). (**D**) GO enrichment analysis of the group B genes classified in **Figure 2A**. (**E**) GO enrichment analysis of the group C genes classified in **Figure 2A**. The enriched categories shown were based on a cutoff p-value of false discovery rate (FDR) < 0.05. (**F**) Scatterplots comparing gene expression levels between Day 0 macrophages and Day 7 CC5 cells (left panel) or Day 7 CC6 cells (right panel). (**G**) RNA microarray analysis of representative microglia-specific enriched genes. Data are shown as fold-change versus Day 0 macrophages. *** FDR p vale < 0.001. Representative MEGs are highlighted in red and representative CEGs in blue. Dashed line indicates a two-fold change. CEG, cortex-enriched genes; MEG, macrophage-enriched genes; GO, gene ontology.

4 Supplementary Figure 3

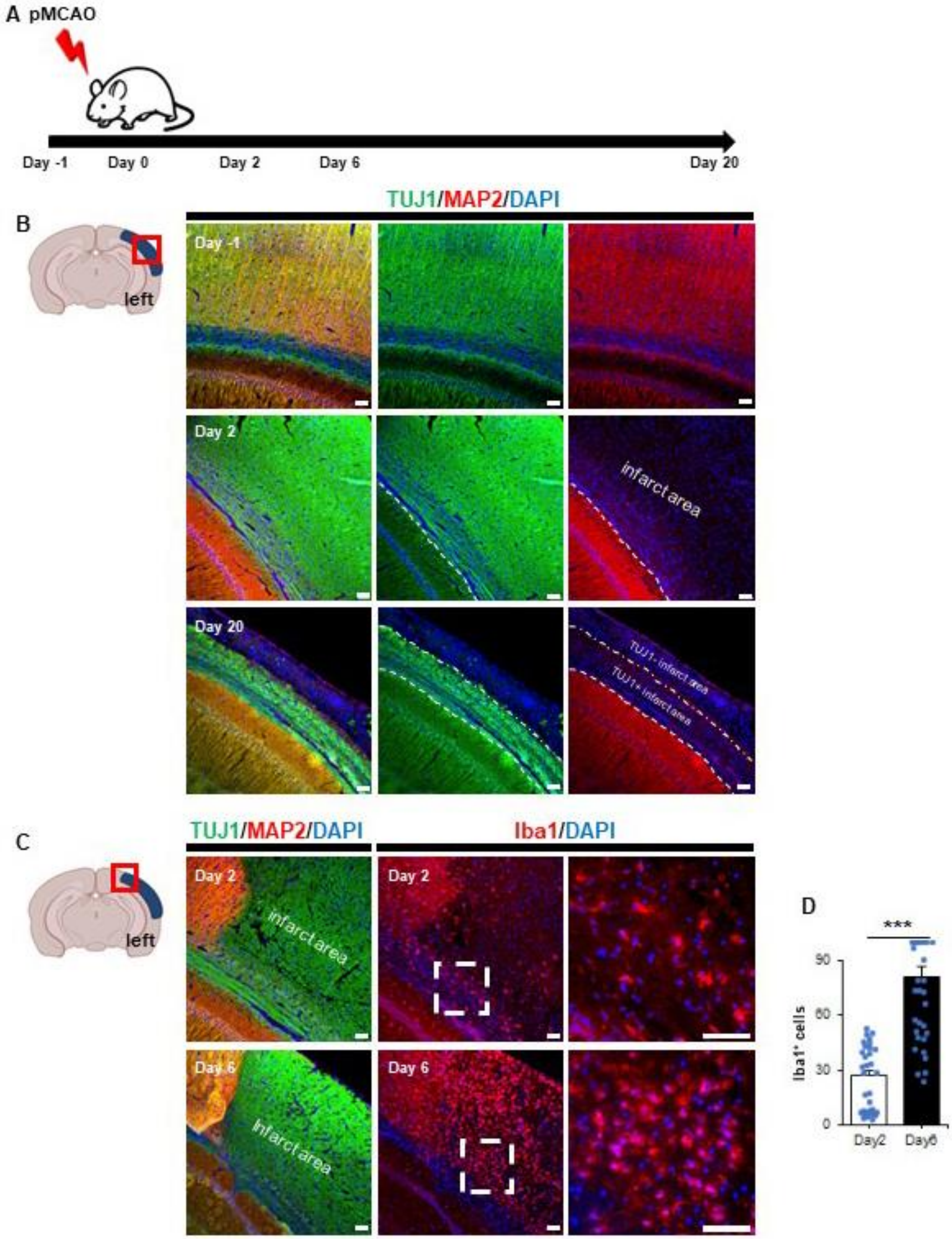

Supplementary Figure 3. Histological changes over time in infarct area of mice. Related to Figure 3.

(**A**) Schematic diagram showing the natural history of mice with permanent MCAO (pMCAO). Mice were sacrificed on days -1, 2, 6, and 20 for immunostaining. (**B**) Immunostaining for TUJ1 and MAP2 1 day before MCAO and 2 and 20 days after MCAO. One day before infarction, the normal cortical tissue indicates positive immunoreactivity for TUJ1 and MAP2 (upper panels). Two days after infarction, the cortical structures are damaged and MAP2 immunoreactivity is lost, but TUJ1 immunoreactivity is retained (middle panels). Twenty days after infarction, the cortex is contracted and TUJ1 immunoreactivity is lost in the outer infarct area (lower panels). Representative images (**C**) and quantitative analysis (**D**) show a significant increase in macrophage counts in the infarct area on Day 6 compared to Day 2. The total number of Iba1+ cells in the infarct area is quantified based on 10 randomly selected view fields in the infarct area for each sample. Mean ± SEM, n = 3 independent experiments per n = six to seven views. *** $p < 0.001$, Scale bar, 50 μm. MCAO, middle cerebral artery occlusion; TUJ1, beta III tubulin; MAP2, microtubule associated protein 2; SEM, standard error of mean.

5 Supplementary Figure 4

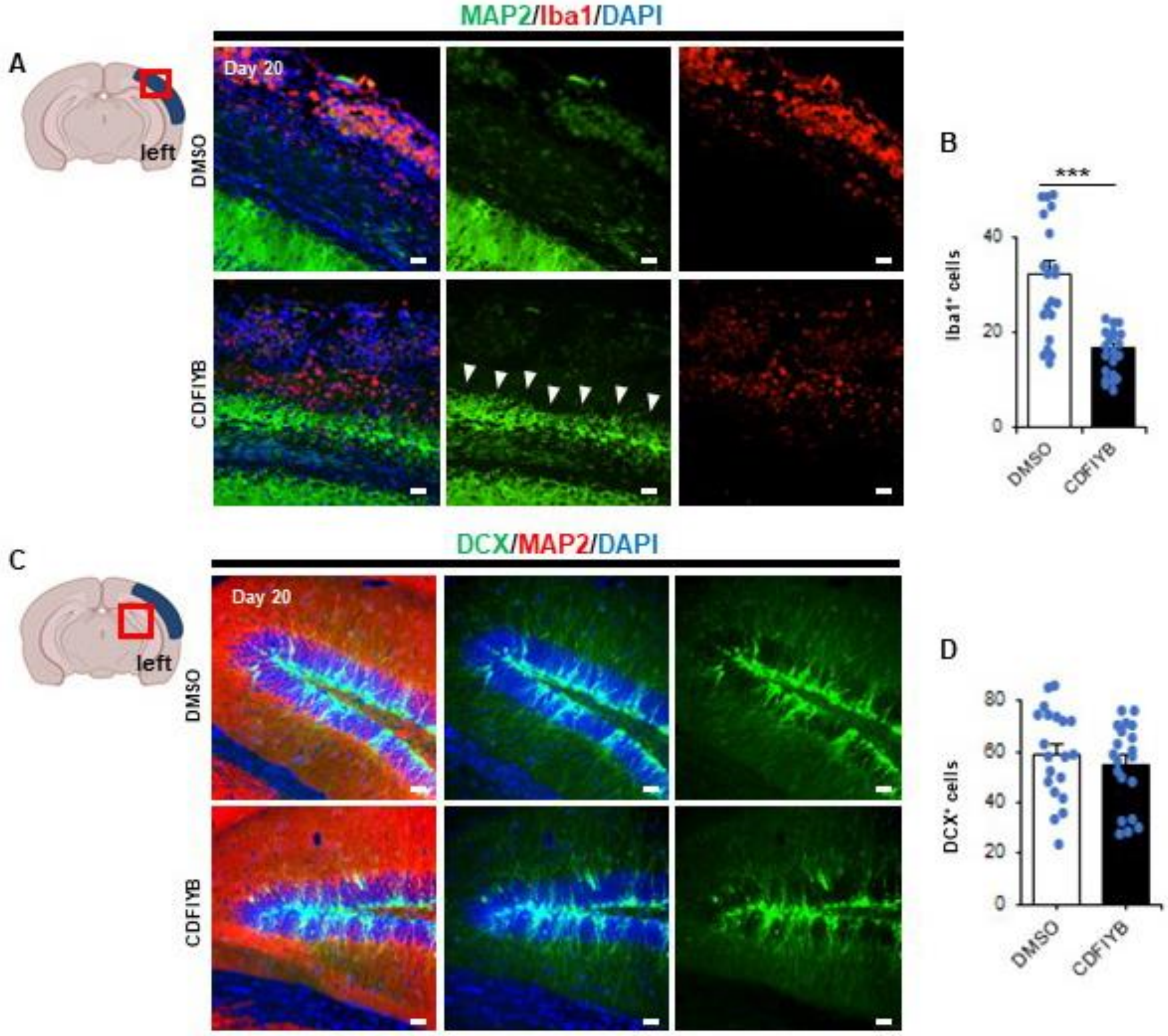

Supplementary Figure 4. Further analysis of cerebral infarct area in mice treated with six drugs. Related to Figure 3.

Representative images (**A**) and quantitative analyses (**B**) showing that compared to the vehicle control, the six drugs significantly decreased the macrophage number (Student's t test, ***$p < 0.001$, n = 3 mice). Arrowheads indicate a new neuronal layer generated by the treatment with the six drugs. Scale bar, 50 µm. The total number of Iba1+ cells in the infarct area was quantified based on 6~7 randomly selected view fields in the infarct area for each sample. Representative images (**C**) and quantitative analyses (**D**) showing that compared to the vehicle control, small-molecule treatment did not significantly alter the number of newborn neurons in the hippocampal dentate gyrus (n = 3 mice). Scale bar, 50 µm. The total number of DCX+ cells in the left hippocampal dentate gyrus is quantified based on 6~7 randomly selected view fields for each sample. DCX, doublecortin.
